# The creation and validation of predictive models to assess the risk of unfavorable outcomes following hybrid total arch repair for Stanford type A aortic dissection

**DOI:** 10.1186/s12872-023-03642-9

**Published:** 2023-12-10

**Authors:** Xinyi Liu, Xing Liu, Yuehang Yang, Ai Zhang, Jiawei Shi, Huadong Li, Junwei Liu, Xionggang Jiang, Zhiwen Wang

**Affiliations:** 1 Department of Cardiovascular Surgery, Union Hospital, Tongji Medical College, Huazhong University of Science and Technology, Wuhan, 430022 Hubei China; 2 Department of the First Specialty Internal Medicine, The Hubei Armed Police Corps Hospital, Wuhan, 430061 Hubei China

**Keywords:** Stanford type A Aortic Dissection, In-hospital compound adverse events, Nomogram

## Abstract

**Background:**

The objective of this study was to develop and validate a nomogram for the individualized prediction of adverse events in patients with Stanford type A aortic dissection (TAAD) undergoing hybrid total aortic arch repair.

**Methods:**

From April 2019 to April 2022, we conducted a comprehensive review of the medical records of Stanford type A aortic dissection patients who underwent hybrid total aortic arch repair surgery at our hospital. Patients were separated into two groups based on whether or not a composite adverse event occurred following surgery. Using univariate and multivariate analyses of logistic regression, the prediction model was created. Construct risk prediction models utilizing nomograms and evaluate their precision, discrimination, and clinical utility.

**Results:**

Age, platelets, serum blood urea nitrogen, and ascending aortic diameter were the variables included in the nomogram by univariate and multivariate analysis. The risk model performed well in internal validation, with an area under the curve (AUC) of 0.829. The calibration curve demonstrated good agreement between predicted and actual probabilities (Hosmer-Lemeshow test, *P* = 0.22). Clinical decision analysis curves demonstrate predictive nomograms’ clinical utility.

**Conclusion:**

This study created and validated a nomogram for predicting the risk of composite endpoint events in TAAD patients undergoing hybrid total aortic arch repair. The nomogram can help determine the severity of a patient’s condition and provide a more personalized diagnosis and treatment.

**Supplementary Information:**

The online version contains supplementary material available at 10.1186/s12872-023-03642-9.

## Introduction

Aortic dissection (AD) is a catastrophic cardiovascular disease that results in significant mortality and severe postoperative complications, particularly in patients with Stanford type A aortic dissection (TAAD) [1]. A single-stage hybrid total aortic arch repair(HAR) includes all three of the following procedures: replacement of the ascending aorta, debranching of the arch vessels, and repair of the thoracic aorta endovascularly. Due to the fact that it does not necessitate a deep hypothermic circulatory arrest(DHCA), it has seen widespread application in TAAD patients who are at an extremely high risk of undergoing routine repair [2, 3]. Although avoiding DHCA theoretically provides TAAD patients with theoretical organ protection, postoperative cardiopulmonary vascular complications and postoperative all-cause mortality remain clinical challenges [4]. If the outcomes of preoperatively related exams can predict the prevalence of postoperative adverse events, more targeted diagnosis and treatment measures can be implemented prior to surgery. As a consequence of this, we are in desperate need of specific predictive methods for the purpose of this. It has been suggested that the nomogram is a helpful tool for producing an easy-to-understand visual graph of a numerical predictive model that quantifies the risk of a clinical outcome [5].

The purpose of this study was to develop and validate a nomogram for the individualized prediction of adverse events in TAAD patients undergoing HAR.

## Methods

**Fig. 1:**
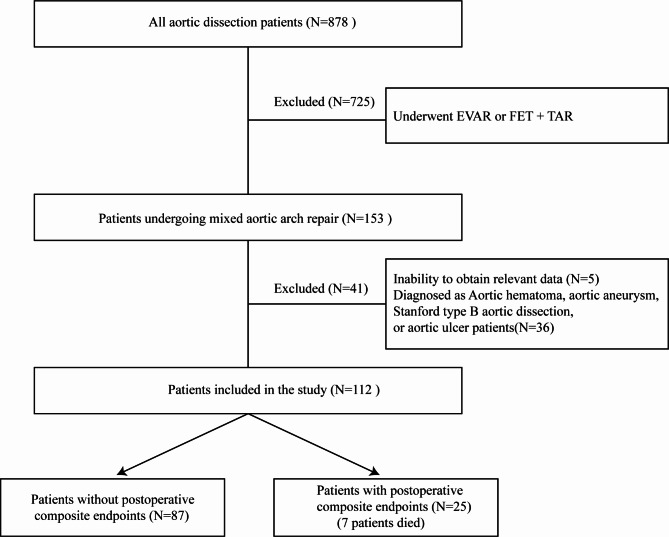
Process of choosing and grouping patients is shown in a flowchart

From April 2019 to April 2022, we retrospectively reviewed surgically treated aortic dissection patients at our hospital. In all patients, computed tomography (CTA) was performed to confirm the diagnosis. To evaluate cardiac function, transthoracic echocardiography is used. Our study exclusion criteria were as follows: (1) aortic hematoma, aortic aneurysm, aortic ulcer, or Stanford type B aortic dissection patients. (2) underwent total arch replacement (TAR) and frozen elephant trunk (FET) surgical procedures. (3) underwent endovascular aortic repair (EVAR). (4) Inability to obtain relevant data. Finally, we included 112 patients with TAAD who all underwent hybrid total aortic arch repair (Fig. 1). We gathered information on demographics, preoperative information, and postoperative results. This study was approved by the ethics committee of Wuhan Union Hospital, Huazhong University of Science and Technology (UHCT22975) and complied with the World Medical Association Code of Ethics (Declaration of Helsinki) adopted in 1975.

### Study composite endpoint

The study’s composite endpoints were postoperative mortality and in-hospital adverse events. Postoperative mortality was defined as death from any cause within thirty days of surgery. The occurrence of any of the following events in the hospital following surgery was considered to be a postoperative in-hospital adverse event. (1) Postoperative stroke was defined as new brain injury that was clinically or radiographically evident after the procedure. (2) After-surgery paraplegia, less than or equal to grade 3 lower limb myodynamia. (3) Severe pulmonary function impairment occurs after surgery, and the duration of ventilator breathing support via tracheal intubation exceeds seven days. (4) Renal failure resulting from surgery necessitates hemodialysis. (5) Requiring the support of extracorporeal membrane oxygenation (ECMO). (6) Unplanned reoperation due to hemostasis or complications related to severe malperfusion.

### Surgical indication

HAR is presently one of the common surgical techniques used by TAAD in our hospital. HAR is the most widely utilized treatment for this condition in the following situations: (1) By comprehensively evaluating the patient’s aortic anatomy, such as the proximal and distal landing zones of the aorta, serious aortic calcification, severe aortic intimal tears, and anatomical false lumen affecting the distal section of the descending aorta. (2) The risk of the DHCA procedure is higher in elderly patients with multiple complications.

### Surgical techniques

At our hospital, three fixed chief physicians with the same surgical experience performed HAR for TAAD. Our HAR has two components: open repair and endovascular repair. It is a single stage procedure performed in a hybrid operating room.

In the open repair section, we perform a median sternotomy from the suprasternal fossa. By separating the aortic arch branches, the aortic arch is visualized. The right femoral artery and right atrium were then cannulated to perform cardiopulmonary bypass (CPB). Surgery is performed under moderate hypothermia circulatory arrest (MHCA). The aorta was clamped after the temperature was lowered to 28–32 °C. According to the situation, the aortic root dissection was completed, the false lumen thrombus was removed, and the aortic root was reinforced. Three brachiocephalic vessels were separately clamped. left common carotid artery and innominate artery cannulation for bilateral cerebral perfusion. The four-branched artificial blood vessel and the distal end of the ascending aorta were anastomosed end-to-end. Pull down the four-branched artificial blood vessel after that and connect it to the proximal autologous blood vessel by performing an end-to-end anastomosis. Open the trunk of the artificial blood vessel and restore the temperature to allow the heart to automatically rebeat. By antegrade perfusion of the furthest branch of the four-branched artificial blood vessel, the left common carotid, the left subclavian, and the innominate arteries were sequentially rebuilt. After protamine was given to neutralize the heparin, CPB was stopped, and wound hemostasis was achieved.

The original right femoral artery cannula’s incision was used to retrogradely implant the stent during the endovascular repair procedure. To complete the arch repair, the proximal stent was anchored to the artificial vessel. Angiograms revealed that the thoracic aorta’s false lumen was free of any endoleaks or contrast material. After adequate hemostasis and chest closure, the femoral artery incision was stitched up, and the procedure was then finished.

### Statistical analysis

Categorical variables were expressed as frequencies and percentages in the patient’s baseline characteristics, while continuous variables were expressed as the mean, standard deviation or median, and interquartile range (IQR). The chi-square test, or Fisher’s exact test, was used to analyze categorical variables. An independent t-test was used for continuous variables with normal distribution. Use the Mann-Whitney U test for continuous variables that are not normally distributed. A univariate logistic regression analysis was used to determine the significance of each variable. Variables with *P* < 0.05 were entered into a multiple logistic regression analysis, with variables with *P* < 0.05 considered possible predictors.

A nomogram for predicting adverse events after HAR in patients with TAAD was created based on the results of the final regression analysis. We plotted the ROC curve and measured the area under the curve (AUC) to assess the nomogram’s discriminative performance. Combined with the Hosmer-Lemeshow (HL) test, a calibration curve was generated for calibration. Furthermore, the calibration was carried out using a calibration curve and 1,000 bootstrap resamples for internal validation in order to assess their predictive accuracy. Finally, to evaluate the clinical usefulness of the generated nomograms, we conducted clinical decision curve analysis (DCA) and clinical impact curve analysis. Data analysis was carried out using R software (version 4.2.1) and SPSS Statistics for Windows, v27.0 (SPSS, USA). All tests were two-tailed, and *P* < 0.05 was considered significant.

## Result

112 patients who were diagnosed with TAAD and underwent HAR were included in this study. A postoperative composite endpoint occurred in 25 patients, of whom 7 passed away within 30 days of surgery, 2 experienced postoperative stroke, 1 experienced postoperative paraplegia, 8 experienced severe pulmonary function impairment, and 7 required hemodialysis due to postoperative renal failure. The baseline characteristics of all patients are shown in Table 1 and Supplementary File 1.

**Table 1:** Preoperative characteristics of the study population

Variable	Non-endpoint	endpoint	*p* value
N	87	25	
**Patient characteristics**			
Age (years)	56(51–59)	63(55–67)	0.003
Male	68(78.2%)	20(80.0%)	0.892
Height (cm)	169.3 ± 5.5	168.2 ± 7.5	0.409
Weight (Kg)	72.1 ± 11.4	73.1 ± 14.9	0.685
BMI	25.1 ± 3.3	25.7 ± 3.78	0.418
**Medical history**			
Hypertension	66(75.8%)	18(72.0%)	0.942
Diabetes	14(16.1)	3(12.0%)	0.565
Coronary artery disease	7(8.1%)	3(12.0%)	0.489
Chronic kidney disease	0(0%)	3(12.0%)	0.046
Cerebrovascular event	0(0%)	4(16.0%)	0.016
Alcohol consumption	20(23.0%)	5(20%)	0.802
Smoking	36(41.4%)	9(36.0%)	0.829
**Clinical features at presentation**			
Heart rate (b.p.m)	81(70–90)	63(55–67)	0.003
SBP (mmHg)	138.9 ± 22.4	136.7 ± 21.1	0.623
DBP (mmHg)	79.3 ± 16.3	77.9 ± 15.1	0.678
**Laboratory parameters**			
RBC (10^9^ /L)	4.2(3.8–4.5)	3.88(3.4–4.3)	0.016
HCT (fl)	37.9 ± 5.7	34.7 ± 5.5	0.008
Platele (10^9^/L)	164(135–202)	132(104–159)	0.003
WBC (10^9^/L)	9.7(7.9–13.4)	8.9(7.6–11.2)	0.351
Lymphocyte percentage (%)	10.2 ± 6.1	10.3 ± 5.9	0.956
Haemoglobin (g/L)	126.7 ± 19.1	116.6 ± 19.3	0.015
Serum albumin (g/L)	36.3 ± 3.9	34.1 ± 4.8	0.017
Creatinine (μmol/L)	89.2 ± 54.3	150.4 ± 158.24	0.007
Serum urea nitrogen (mmol/L)	6.7 ± 2.9	9.7 ± 5.3	< 0.001
Admission glucose (mmol/L)	7.1 ± 3.1	2.3 ± 2.3	0.761
APTT (s)	39.1(34.6–42.3)	38.2(34.1–40.6)	0.404
INR	1.12(1.05–1.19)	1.15(1.08–1.25)	0.061
CK-MB (ng/ml)	2.1 ± 3.8	2.4 ± 2.3	0.761
**Imaging parameters**			
EF (%)	62.6 ± 4.1	60.8 ± 4.3	0.036
Pericardial effusion	32(36.8%)	6(24.0%)	0.367
Aortic sinus inner diameter (cm)	3.9 ± 0.6	4.1 ± 0.6	0.155
Ascending aorta diameter (cm)	4.6 ± 0.7	5.1 ± 0.8	0.003
Distal inner diameter of aortic arch (cm)	3.4 ± 0.4	3.5 ± 0.5	0.196
Proximal inner diameter of descending thoracic aorta (cm)	3.9(3.5–4.2)	4.0(3.3–4.4)	0.985

Values are expressed as the means ± standard deviations, numbers, or medians (interquartile range, IQR). A *P* value for a linear trend of < 0.05 is statistically significant. BMI, body mass index; SBP, systolic blood pressure; DBP, diastole blood pressure; RBC, red blood cell; HCT, haematocrit; WBC, white blood cell; APTT, activated partial thromboplastin time; INR, International Normalized Ratio; CK-MB, creatine kinase MB isoenzyme; EF, ejection fraction

We began with a univariate logistic regression analysis of the variables in Table 1. We began with a univariate logistic regression analysis of the variables in Table 1. By comparing the two sets of data, we discovered that age (OR = 1.07, 95% CI 1.01 to 1.13, *p* = 0.02), platelets (OR = 0.98, 95% CI 0.97 to 0.99, *p* = 0.004), hemoglobin (OR = 0.97, 95% CI 0.95 to 0.99, *p* = 0.02), serum albumin (OR = 0.89, 95% CI 0.81 to 0.98, *p* = 0.02), creatinine(OR = 1.01, 95% CI 1.00 to 1.02, *p* = 0.03), serum urea nitrogen(OR = 1.23, 95% CI 1.07 to 1.41, *p* = 0.004), CK-MB(OR = 0.89, 95% CI 0.80 to 0.99, *p* = 0.04), ascending aorta diameter(OR = 2.37, 95% CI 1.28 to 4.38, *p* = 0.006), showed statistical difference. The above eight variables were then subjected to a multivariate logistic regression model analysis. We identified age, platele, serum urea nitrogen, and ascending aorta diameter as independent predictors of composite endpoints after HAR(Table 2).

**Table 2:** Preoperative variables and their association to the composite endpoint

Variable	Univariable model		Multivariable model	
	OR (95% CI)	*p* value	OR (95% CI)	*p* value
Age (years)	1.07(1.01–1.13)	0.02	1.07(0.99–1.15)	0.04
Male	1.08(0.39–3.04)	0.88		
Height (cm)	0.97(0.97–1.04)	0.41		
Weight (Kg)	1.01(0.97–1.04)	0.68		
BMI	1.05(0.93–1.18)	0.41		
Hypertension	0.94(0.36–2.5)	0.91		
Diabetes	0.66(0.19–2.3)	0.51		
Coronary artery disease	1.87(0.51–6.96)	0.35		
Alcohol consumption	0.84(0.30–2.33)	0.74		
Smoking	0.85(0.36-2.00)	0.85		
Heart rate (b.p.m)	0.99(0.96–1.02	0.51		
SBP (mmHg)	0.99(0.97–1.01)	0.62		
DBP (mmHg)	0.99(0.97–1.02)	0.68		
RBC (109 /L)	0.70(0.42–1.16)	0.17		
HCT (fl)	0.91(0.83–0.98)	0.11		
Platele (109/L)	0.98(0.97–0.99)	0.004	0.98(0.97–0.99)	0.02
WBC (109/L)	0.94(0.84–1.07)	0.37		
Lymphocyte percentage	1.01(0.93–1.07)	0.96		
Haemoglobin (g/L)	0.97(0.95–0.99)	0.02	0.99(0.96–1.03)	0.64
Serum albumin (g/L)	0.89(0.81–0.98)	0.02	1.03(0.89–1.19)	0.71
Creatinine (μmol/L)	1.01(1.00-1.02)	0.03	1.01(0.99–1.02)	0.48
Serum urea nitrogen (mmol/L)	1.23(1.07–1.41)	0.004	1.24(1.09–1.41)	0.002
Admission glucose (mmol/L)	0.99(0.86–1.15)	0.92		
APTT (s)	0.96(0.89–1.03)	0.26		
INR	2.18(0.89–9.76)	0.06		
CK-MB (ng/ml)	1.02(0.90–1.15)	0.76		
EF (%)	0.89(0.80–0.99)	0.04	0.92(0.81–1.04)	0.18
Pericardial effusion	0.61(0.24–1.54)	0.29		
Aortic sinus inner diameter (cm)	0.75(0.54–0.93)	0.25		
Ascending aorta diameter (cm)	2.37(1.28–4.38)	0.006	2.38(1.08–5.23)	0.03
Distal inner diameter of aortic arch (cm)	1.86(0.73–4.8)	0.19		
Proximal inner diameter of descending thoracic aorta (cm)	1.22(0.63–2.35)	0.97		

OR (95% CI) indicates the proportional odds ratio with 95% confidence interval; for other abbreviations, please see Table 1. *P* value < 0.05 was considered statistically significant

**Fig. 2:**
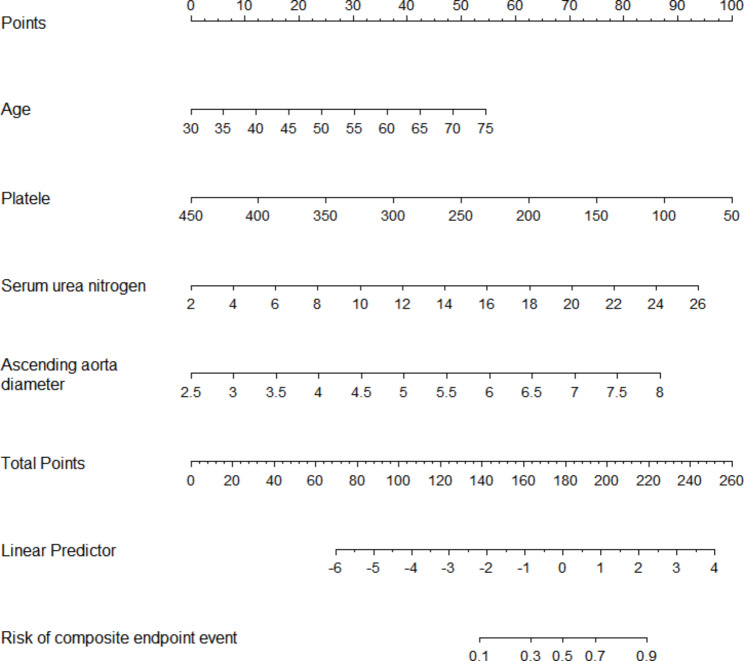
A nomogram for evaluating the risk of a composite endpoint event after hybrid aortic repair in people with Stanford type A aortic dissection. The four individual scores can be added up to create the total score, which can then be used to calculate the risk of composite endpoint corresponding probability of occurrence.

A nomogram was constructed to predict the probability of a composite endpoint event, which included the four independent risk factors mentioned above (Fig. 2). The four individual scores can be added up to create the total score, which can then be used to calculate the risk of a composite endpoint and its corresponding probability of occurrence.

**Fig. 3:**
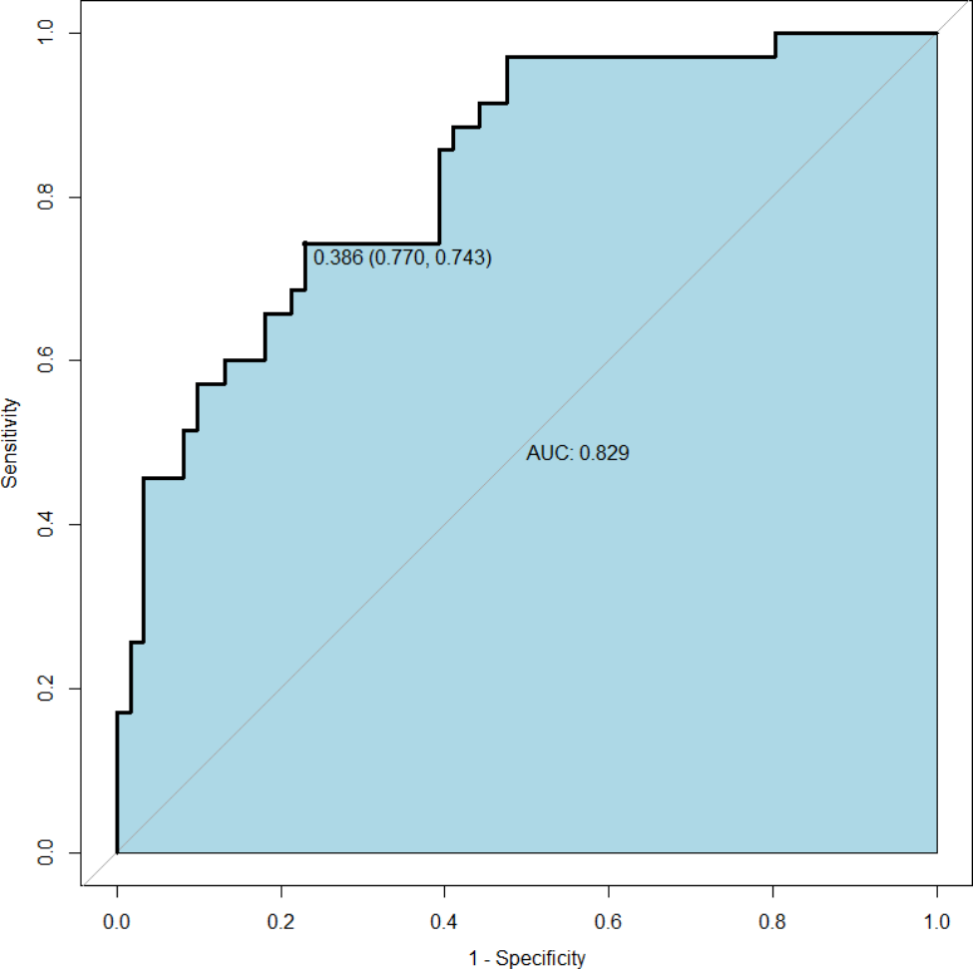
The ROC curve used to evaluate the discriminative performance of the model, the area under the curve AUC was 0.829, and the optimal cutoff point suggested a sensitivity of 77.0% and a specificity of 74.3%.

**Fig. 4:**
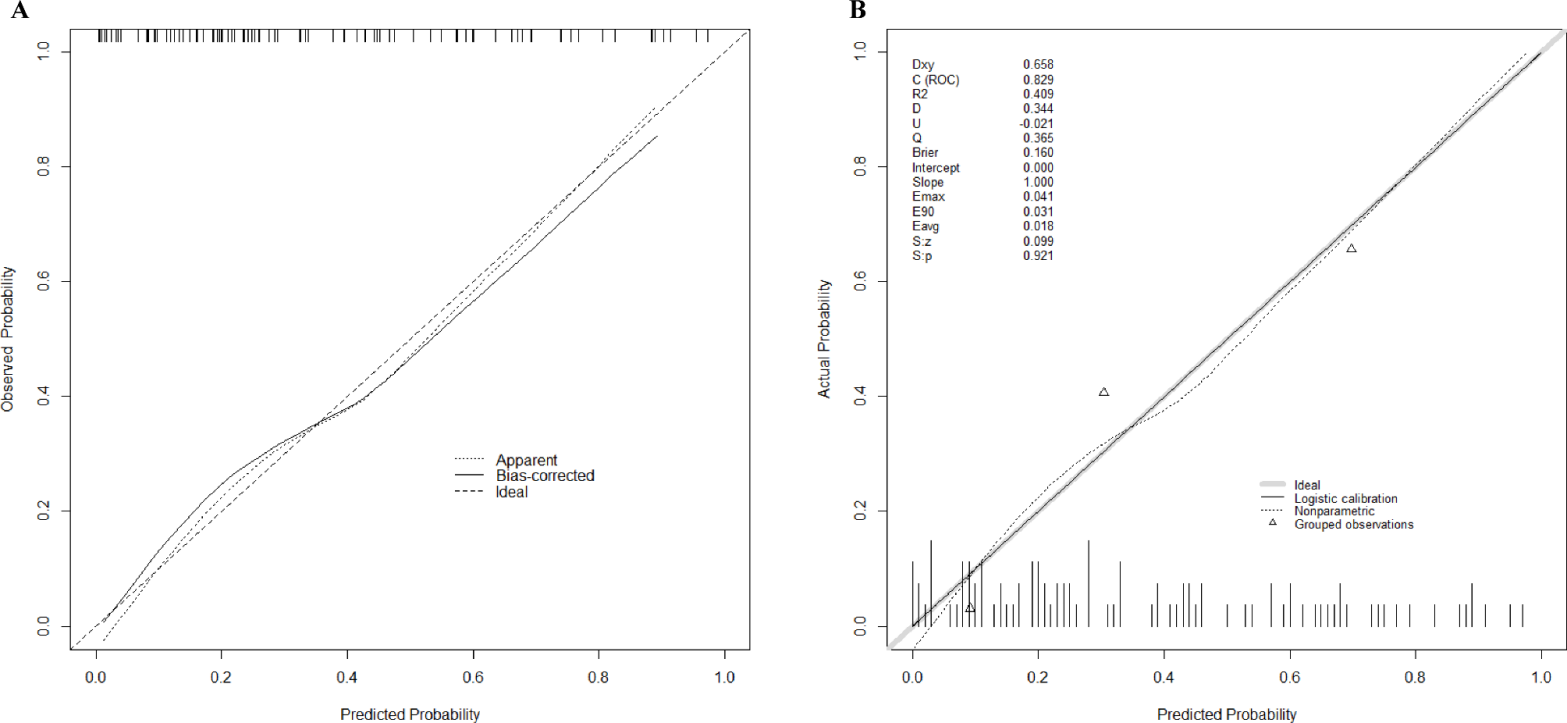
(**A**) The calibration curve for the prediction model shows the degree of agreement between the predicted risk and the actual outcome, with the predicted risk on the x-axis and the actual outcome on the y-axis. (**B**) Brier score and other correlation probability calibration values are displayed, and indicate a good probability calibration effect.

Plot the ROC curve to evaluate the resulting model, which includes the four variables listed above (Fig. 3). Calculate the concordance index (C-index), resulting in a C-index of 0.829 (95% CI 0.747 to 0.911) and a bias-corrected C-index of 0.801. The ROC curve shows that the model has good discriminative power with an AUC of 0.829. Predicted probabilities of postoperative composite endpoints are in good agreement with actual observations, according to calibration curves (Fig. 4A).

A good model fit was demonstrated by the Hosmer-Lemeshow test, which revealed no statistical significance (*p* = 0.22). The Brier score is 0.160, indicating a good probability calibration effect (Fig. 4B).

**Fig. 5:**
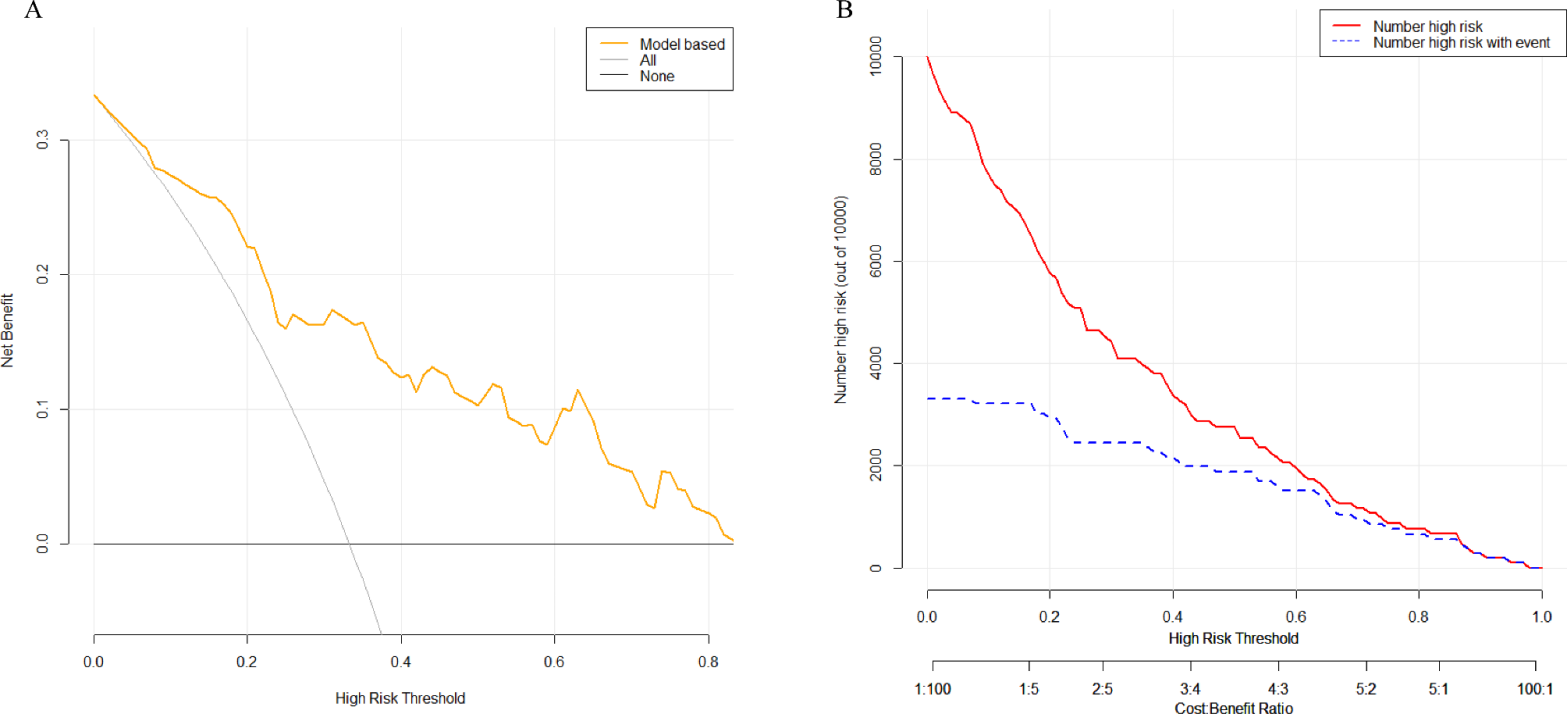
(**A**) Clinical decision curve analysis of predictive models. The Y-axis represents the net benefit, the orange line depicts the predictive model, the “All” curve represents all intervention, the “NO” curve represents no intervention at all, and the curve shows that the model outperforms both “intervention” and “no intervention” treatment strategies. (**B**) The red line represents the predictive model, and the blue dashed line represents the actual data, in the clinical impact curve of the predictive model. The graph indicates that the predictive model is clinically useful.

The predictive model’s clinical decision curve analysis and clinical impact curve analysis (Fig. 5A and B) revealed that it has a good clinical application value, which strengthened our model.

## Discussion

In this study, we developed and tested a nomogram to predict the risk of in-hospital adverse events in TAAD patients undergoing HAR. Demographics, laboratory findings, and imaging characteristics were all included in the nomogram. The nomogram has good discrimination and calibration performance as well as clinical application value.

HAR is currently an attractive option for treating TAAD. Theoretically, surgical trauma and risk can be decreased because deep hypothermic circulatory arrest is not necessary and myocardial ischemia can be decreased by reducing the duration of the aortic cross-clamp [6–8]. However, a number of clinical prognosis studies have noted that after HAR, there may be a higher risk of neurological adverse events, such as new brain injury, stroke, etc. [9, 10]. This might be connected to the aortic arch surgery and the stent release that caused the atherosclerotic plaque to rupture [6, 11]. Second, there are additional postoperative adverse events that cannot be disregarded, including the need for prolonged tracheal intubation and ventilator-assisted breathing due to new-onset lung injury and the requirement for hemodialysis due to new-onset renal failure following HAR surgery. Because the aforementioned complications from HAR can significantly raise patients’ risk of being readmitted to the hospital and can negatively impact their quality of life. The composite endpoint events of our study were therefore the aforementioned adverse prognostic events and all-cause mortality within 30 days following surgery in the hopes that the developed nomogram could comprehensively predict the adverse reactions of patients following HAR surgery.

Age, platelets, serum urea nitrogen, and preoperative ascending aortic diameter were the four variables we used to build a predictive model in this study. Among them, serum urea nitrogen and age are crucial independent prognostic factors for composite endpoint events in TAAD patients receiving HAR. On the one hand, the glomerular filtration rate declines and organ function degenerates as people age, raising serum urea nitrogen levels. On the other hand, TAAD can reduce the excretion of serum urea nitrogen by increasing the activity of the sympathetic nervous system and the renin-angiotensin-aldosterone system [12, 13]. Elevated blood urea nitrogen levels may exacerbate the progression of TAAD via hemodynamic factors. According to Liu and colleagues, among patients with acute aortic dissection, admission serum urea nitrogen levels were an independent predictor of in-hospital mortality [14]. This judgement is also supported by our findings.

The coagulation process depends on platelets, which can also release a number of cytokines that are involved in the body’s inflammatory response [15]. Our study showed that preoperative platelet count was a predictor of postoperative composite endpoint events. According to Yu et al., a low preoperative platelet count is an independent predictor of postoperative neurological complications, and platelet consuming coagulation disorders may be to blame [16]. Li et al. have also pointed out that platelet count is associated with in-hospital mortality after TAAD, which may be related to the intimal tear of the aortic dissection leading to the release of subendothelial tissue factor and initiation of the coagulation cascade [17].

In addition, preoperative ascending aortic diameter was found to be a predictor of adverse events after HAR in TAAD patients. This may be the case because, as the dissection tear enlarges the ascending aorta, the elastin fibers in the aortic wall begin to break down [18]. This results in decreased vascular compliance, which in turn leads to a worse prognosis. Simultaneously, the CTA examination is a routine examination prior to TAAD surgery, and the inner diameter of the ascending aorta is also easily obtained data, implying that it has broad clinical application prospects.

There are some limitations to this study. First and foremost, as a retrospective study, this study has inherent limitations. Second, this model’s misdiagnosis rate was still present. Third, this study lacks short to intermediate term follow up data. Finally, in this study, the nomogram design is based on our single-center data and has only been internally validated for accuracy. It is necessary to validate the results at other centers in the future.

## Conlusion

In conclusion, this study created and validated a nomogram for predicting the risk of composite endpoint events in TAAD patients undergoing HAR. The nomogram can help determine the severity of a patient’s condition and provide more personalised diagnosis and treatment.

### Electronic supplementary material

Below is the link to the electronic supplementary material.

**Supplementary Material 1: Supplementary File 1**. Supplementary data for the study population

## Data Availability

The original contributions presented in the study are included in the article material, further inquiries can be directed to the corresponding author.
