# Supplementary material for: The creation and validation of predictive models to assess the risk of unfavorable outcomes following hybrid total arch repair for Stanford type A aortic dissection
Source: BMC Cardiovasc Disord. 2023 Dec 10;23:606. doi: 10.1186/s12872-023-03642-9 (PMC10710709; doi:10.1186/s12872-023-03642-9)
Supplement: Supplementary file 1 — Supplementary Material 1: Supplementary File 1. Supplementary data for the study population [file 12872_2023_3642_MOESM1_ESM.docx]

| **Variable** | **Non-endpoint** | **endpoint** | ***p* value** |
| --- | --- | --- | --- |
| N | 87 | 25 |  |
| **Preoperative variables** |  |  |  |
| Pericardial effusion | 31(35.6%) | 7(28.0%) | 0.367 |
| Stroke | 0(0.0%) | 4(16.0%) | 0.016 |
| Chronic liver disease | 2(2.3%) | 0(0.0%) | 0.298 |
| Dialysis | 0(0.0%) | 3(12.0%) | 0.046 |
| Pulmonary embolism | 1(1.1%) | 2(8.0%) | 0.130 |
| History of heart surgery | 0(0.0%) | 2(8.0%) | 0.095 |
| Preoperative CPCR | 0(0.0%) | 0(0.0%) | 1.000 |
| Moderate to severe aortic regurgitation | 34(39.1%) | 12(48.0%) | 0.136 |
| Emergency operation | 51(58.6%) | 16(64.0%) | 0.663 |
| **Intraoperative variables** |  |  |  |
| CPB time(min) | 153.0(124.5-178.0) | 160.0(135.0-185.0) | 0.073 |
| ACCT(min) | 99.0(68.0-115.0) | 101.0(85.0-126.0) | 0.135 |
| Intraoperative RBC transfusion(unit) | 6.0(4.0-7.0) | 6.0(5.0-10.0) | 0.182 |
| Intraoperative plasma transfusion(mL) | 600.0(500.0-775.0) | 600.0(500.0-850.0) | 0.198 |
| Intraoperative PLT transfusion(unit) | 2.0(2.0-3.0) | 2.0(2.0-4.0) | 0.192 |
| Aortic root management | 9(10.3%) | 7(28.0%) | 0.052 |
| David | 0(0.0%) | 0(0.0%) | 1.000 |
| Bentall | 8(9.2%) | 5(20.0%) | 0.131 |
| Wheat | 1(1.1%) | 2(8.0%) | 0.139 |
| CABG | 5(5.7%) | 4(16%) | 0.163 |

**Supplementary File 1. Supplementary data for the study population**

Values are expressed as the numbers or medians (interquartile range, IQR). A *P* value for a linear trend of < 0.05 is statistically significant. CPCR, Cardiopulmonary cerebral resuscitation; CPB, Cardiopulmonary bypass; ACCT, aortic cross clamp time; RBC, Red blood cell; PLT, Platelet; CABG, Coronary Artery Bypass Grafting.

**Supplementary variable univariate model**

| **Variable** | **Univariable model** | |
| --- | --- | --- |
|  | **OR (95% CI)** | ***p* value** |
| Pericardial effusion | 0.61(0.24-1.54) | 0.299 |
| Stroke |  | >0.999 |
| Chronic liver disease |  | >0.999 |
| Dialysis |  | >0.999 |
| Pulmonary embolism |  | >0.999 |
| History of heart surgery |  | >0.999 |
| Moderate to severe aortic regurgitation | 1.36(0.89-2.07) | 0.158 |
| Emergency operation | 1.33(0.56-3.16) | 0.517 |
| CPB time(min) | 1.08(1.01-1.15) | 0.092 |
| ACCT(min) | 1.01(0.98-1.03) | 0.073 |
| Intraoperative RBC transfusion(unit) | 1.14(0.99-1.31) | 0.064 |
| Intraoperative plasma transfusion(mL) | 1.01(0.98-1.02) | 0.213 |
| Intraoperative PLT transfusion(unit) | 1.17(0.95-1.24) | 0.095 |
| Aortic root management | 3.08(0.79-9.05) | 0.062 |
| David |  | >0.999 |
| Bentall | 2.71(0.86-8.62) | 0.091 |
| Wheat | 5.63(0.56-16.32) | 0.142 |
| CABG | 2.95(0.77-11.28) | 0.114 |

OR (95% CI) indicates the proportional odds ratio with 95% confidence interval. *P* value < 0.05 was considered statistically significant.
